# Effect of an astrocyte calcium exporter on orbitofrontal cortex neuron excitability, astrocyte-synaptic interaction, and alcohol consumption

**DOI:** 10.1016/j.neuropharm.2025.110365

**Published:** 2025-02-13

**Authors:** A.R. Kastner-Blasczyk, S.W. Hester, S.E. Reasons, M.D. Scofield, J.J. Woodward

**Affiliations:** a Department of Neuroscience, Medical University of South Carolina, Charleston, SC, United States; b Department of Anesthesia and Perioperative Medicine, Medical University of South Carolina, Charleston, SC, United States

**Keywords:** Astrocyte, Alcohol, Orbitofrontal cortex, PMCA, Confocal imaging, Electrophysiology

## Abstract

Previous electrophysiology studies show that acute ethanol inhibits firing of orbitofrontal (OFC) cortex neurons while chronic intermittent ethanol (CIE) exposure increases firing accompanied by enhanced ethanol drinking. The acute ethanol inhibition of OFC neuronal firing is mediated by inhibitory glycine receptors and is reduced by expressing a plasma membrane calcium ATPase (PMCA) in OFC astrocytes. In this study, we tested the effects of astrocyte PMCA on CIE-induced increases in excitability and alcohol consumption and the physical interaction between OFC astrocytes and neurons. CIE increased neuronal firing in male mice as compared to Air controls while PMCA itself increased firing in Air control male mice. In contrast, PMCA reduced CIE-mediated hyperexcitability of firing in females. CIE did not affect OFC astrocyte size in control or PMCA male mice but increased astrocyte size in female mice. Similar to spiking, PMCA and CIE both increased the number of GluA1 containing synapses within the vicinity of virally labeled astrocytes in male mice but had differential effects in females. The astrocytic interaction with GluA1 labeled synapses was not affected by CIE treatment in male or female control mice, but there was a treatment-dependent effect of PMCA in male mice. CIE increased alcohol consumption in control but not PMCA male mice and had no effect on drinking in female mice. Lastly, OFC astrocyte PMCA expression had no effect on behavioral measures of locomotion, anxiety, spontaneous alternation, or spatial memory. These findings reveal important sex-dependent differences in the physiological, structural and behavioral actions of OFC astrocytes.

## 1. Introduction

Alcohol use disorder (AUD) is characterized by frequent and excessive consumption of alcohol in a compulsive manner often to avoid the negative affective state that arises during withdrawal (American Psychiatric Association, 2022). Historically, much of the research into the effects of acute and chronic alcohol exposure on brain function focused on dopaminergic reward pathways and circuitry involved in positive (mesolimbic pathway and ventral striatum) and negative (extended amygdala) effects of alcohol consumption. More recently, these studies have been extended to cortical regions including the orbitofrontal cortex (OFC) that are involved in regulating higher order actions such as choice behavior and reward seeking (Koob and Volkow, 2010) that contribute to the preoccupation/craving for alcohol. For example, studies have demonstrated an important role of the OFC in goal-directed behavior, reversal learning, and assigning value to rewards (Bracht et al., 2021; Noonan et al., 2012; Moorman, 2018; Gremel and Costa, 2013; Cazares et al., 2021; Erickson et al., 2021). Furthermore, results from other studies suggest that the OFC may be important for the motivation to seek alcohol, and in the dependence-induced escalation in drinking (Hernandez and Moorman, 2020; den Hartog et al., 2016). Clinical studies in individuals with AUD report hypoactivation of the lateral OFC (lOFC) at rest but hyperactivation in the presence of alcohol-related visual cues that are positively correlated with the reported strength of craving (Myrick et al., 2004; Seo et al., 2011). In preclinical studies, chronic intermittent ethanol (CIE) exposure induces dependence in C57BL/6J mice that is accompanied by an increase in alcohol consumption (Griffin, 2014; Becker and Lopez, 2004).

Previous electrophysiological studies from this laboratory have demonstrated that acute ethanol inhibits the evoked firing of OFC neurons, while CIE increases OFC neuronal excitability for up to 2 weeks post withdrawal accompanied by tolerance to acute ethanol inhibition (Badanich et al., 2013; Nimitvilai et al., 2016). The acute ethanol-induced inhibition of OFC firing was shown to be mediated by release of glycine from OFC astrocytes and the inhibition could be reduced via expression of a plasma membrane calcium ATPase (PMCA) in astrocytes (Nimitvilai-Roberts et al., 2021). In addition to ongoing studies defining the brain regions involved in initiating and maintaining excessive alcohol drinking, understanding the role of non-neuronal cells such as astrocytes in these actions has become an important goal of research. Glial cells are the most abundant cell type in the central nervous system and play a major role in synaptic function, development, and the maintenance of extracellular homeostasis (Allen and Barres, 2009; Hansson et al., 2000; Theodosis et al., 2008). Given that astroglia are generally not electrically excitable, elevated intracellular astrocytic calcium levels serve as a biomarker of astrocytic activity (Khakh and McCarthy, 2015; Volterra et al., 2014). Astrocytes respond to neuronal activity with elevations of intracellular calcium which drive many cellular functions (Guerra-Gomes et al., 2017) including release of neuroactive molecules termed gliotransmitters (Guerra-Gomes et al., 2018; Santello et al., 2019; Chai et al., 2017). Depending on the gliotransmitter released, these substances can elevate or suppress synaptic strength and neuronal activity (Allen and Barres, 2009; Blomstrand et al., 1999). Interfering with astrocytic calcium signaling disrupts astrocytic action, including gliotransmitter release and downstream astrocyte mediated alterations in synaptic plasticity that may be altered by chronic drug use (Verkhratsky and Kettenmann, 1996; Khakh and McCarthy, 2015; Erickson et al., 2019). Based on these findings, we hypothesized that repeated episodes of ethanol-induced inhibition of OFC neuron firing drives a homeostatic upregulation of OFC excitability and that blunting astrocyte calcium signaling would prevent this adaptation. Furthermore, as chronic exposure to addictive drugs or alcohol can alter astrocyte size, complexity, and their interaction with neurons (Healey et al., 2020; Siemsen et al., 2019; Kruyer et al., 2019; Scofield et al., 2016), we hypothesized that reducing astrocyte calcium activity would also minimize ethanol-induced changes in astrocyte morphology. These hypotheses were tested by expressing a plasma membrane associated calcium exporter (PMCA) in OFC astrocytes of male and female mice followed by repeated cycles of CIE exposure and withdrawal. The effects of PMCA on CIE-induced changes in OFC neuronal excitability, astrocyte morphology and drinking were assessed by electrophysiological, imaging, and behavioral approaches.

## 2. Materials & methods

### 2.1. Animals

Male and female C57Bl/6J mice (8–9 weeks old) were obtained from Jackson Laboratories (Bar Harbor, ME) and were single-housed in a temperature and light-controlled room (12-h light on and 12-h light off) for at least one week prior to undergoing viral surgery. All animals had free access to food and water for the duration of the study and all guidelines were followed per the NIH Guide for the Care and Use of Laboratory Animals and the Medical University of South Carolina’s Institutional Animal Care and Use Committee.

### 2.2. Mouse viral surgery

Mice underwent stereotaxic surgery and received a bilateral 350 nl infusion of either AAV5-GfaABC1D-LCK-GFP (Addgene #105598) or AAV5-GfaABC1D-mCherry-hPMCA2w/b (Addgene #111568) in the lateral orbitofrontal cortex (AP 2.4 mm, ML ± 1.35 mm, and DV −2.4 mm). The LCK-GFP construct expresses a portion of the LCK Src kinase that allows membrane tethering of GFP and serves as a control for the membrane bound PMCA construct. Mice recovered for at least a week in their homecage prior to undergoing further experimental procedures.

### 2.3. Chronic intermittent ethanol exposure

Mice were exposed to alcohol vapor or air for 4 days a week (16-h on and 8-h off) followed by a 72-h withdrawal period as described in previous studies (Zamudio et al., 2021; Becker and Lopez, 2004). During the CIE procedure, cages were placed in a Plexiglas chamber perfused with alcohol vapor (5.0 L/min) at a concentration between 17 and 21 mg/L. Before each daily CIE exposure, mice were given an intraperitoneal injection (20 mL/kg of body weight) of ethanol (1.6 g/kg) and the alcohol dehydrogenase inhibitor (pyrazole: 1.0 mmol/kg) to maintain BEC levels throughout the ethanol vapor exposure time. Air mice were given injections of saline and pyrazole. Blood ethanol concentrations were obtained from the orbital sinus once weekly and analyzed using an Analox Instrument (Lunenburg, MA) with target levels for males: 200–250 mg/dL and females: 250–300 mg/dL.

### 2.4. Slice electrophysiology

Three days following the last CIE or Air exposure, mice were anesthetized with isoflurane and brain slices were collected for electrophysiology studies as previously described (Nimitvilai et al., 2016). Briefly, brain slices (250 μm) were cut using a vibratome (Leica VT1200S) using a solution containing (in mM); 1.9 KCl, 1.2 NaH_2_PO_4_, 0.4 Ascorbic Acid, 6 MgCl_2_, 0.5 CaCl_2_, 10 Glucose, 25 NaHCO_3_, and 200 Sucrose. Slices were then transferred to an oxygenated chamber filled with artificial cerebral spinal fluid (aCSF) at 34 °C for 30 min and then were maintained at room temperature until use. The aCSF contained (in mM) 125 NaCl, 2.5 KCl, 1.25 NaH_2_PO_4_, 0.4 Ascorbic Acid, 1.3 MgCl_2_, 2 CaCl_2_, 10 Glucose, and 25 NaHCO_3_. Pipettes (~2–4 MΩ resistance) were pulled from borosilicate glass (OD 1.5 mm, ID 1.1 mm, Sutter Instrument) using a Sutter Instrument Model P-97 pipette puller. Pipettes were filled with a K-gluconate internal solution (in mM; 10 KCl, 120 K-gluconic acid, 10 HEPES, 2 MgCl_2_, 2 Na_2_ATP, 0.3 Na_3_GTP, and 1 EGTA) titrated to a pH of ~7.4 with 1 M KOH and adjusted to 260–300 mOsm with sucrose if necessary. During recordings, slices were maintained in a recording chamber perfused with 34 °C aCSF at approximately 2 mL/min. Neurons in the deep layers of the OFC were visualized under infrared light using a Zeiss FS2 microscope and were selected for recording in areas showing robust astrocyte fluorescence (LCK-GFP; PMCA-mCherry). After a gigaohm seal and breakthrough were obtained at −70 mV, the input resistance was recorded and then the amplifier (Axon MultiClamp 700B; Molecular Devices, Union City CA) was switched to current-clamp mode with zero current injection to record the resting membrane potential. Current was then injected as needed to adjust the membrane potential to ~ −70 mV as necessary and a series of current steps (−20 – 190 pA, 750 msec each) was applied. Recordings were filtered at 4 kHz and were acquired at 10 kHz using AxographX software (Axograph, Sydney, Australia). Electrophysiology recordings were analyzed using Easy Electrophysiology software (London, United Kingdom). Action potential (AP) threshold was detected when the slope of the membrane potential exceeded 50 mV/ms. AP characteristics (threshold, height, half-width, rise time) were analyzed from the first current step that generated at least 3 action potentials. The after-hyperpolarization (AHP) magnitude was calculated by subtracting the lowest potential during hyperpolarization from the AP threshold and reported values are the mean of the first three AHP magnitudes recorded. Note that the use of the low chloride containing internal solution generated a liquid junction potential of +16.71 mV. Membrane potentials were not corrected for this value.

### 2.5. Transcardial Perfusions and immunohistochemistry

Seventy-two hours after the final CIE exposure, mice were transcardially perfused with ~20 mL of 1X phosphate-buffered saline (PBS) followed by ~20 mL of 4% w/v paraformaldehyde (PFA) and post-fixed for 24 h before being transferred to 0.1 M phosphate buffered saline (PBS) with 0.02% sodium azide for long-term storage. Sections of the OFC were sliced at 60 μm using a Leica VT1000S vibratome (Buffalo Grove, IL) and immunohistochemistry (IHC) was performed following previously published methods (Siemsen et al., 2023). Four slices per animal were blocked on a shaker for 2 h at room temperature in 0.1 M PBS containing 2% (v/v) TritonX-100 (PBST) and 2% (v/v) normal goat serum (NGS). Slices were then incubated in the PBST + NGS blocking solution containing the primary antibody for 16 h on a shaker at 4 °C. The following day, slices were rinsed with PBST 3 times for 5 min before being incubated in the dark with the secondary antibody for 2 h at room temperature on a shaker. Slices were then rinsed with PBST 3 times for 5 min each and were immediately mounted on Superfrost plus slides with Prolong Gold antifade (ThermoFisher Scientific, Waltham, MA, USA) and stored at 4 °C until imaging occurred. Primary antibodies used were rabbit anti-GluA1 (1:1000; Abcam #31232) and chicken anti-mCherry (1:2000; LifeSpan BioSciences #C204825). Secondary antibodies used were Alexa Fluor 647 goat anti-rabbit (1:1000; Thermo Fisher Scientific #A-21244) and Alexa Fluor 594 goat anti-chicken (1:1000; Thermo Fisher Scientific #A-11042).

### 2.6. Confocal microscopy

Confocal images were acquired with a Leica SP8 confocal microscope (Leica Microsystems Inc., Deerfield, IL) with HyD detectors as previously described (Siemsen et al., 2023). Laser power, gain, zoom, and pinhole size were first optimized to maintain voxel saturation consistency between images. Both the Argon 488 nm and Diode 638 nm laser lines were used in experiments with LCK-GFP to capture LCK-GFP and GluA1 expression, respectively. For experiments with PMCA, the OPSL 552 nm laser and the Diode 638 nm laser lines were used to capture mCherry and GluA1 expression, respectively. In all experiments, virally labeled astrocytes were imaged in layer V of the OFC at 1024 x 1024 frame size, 0.3 μm step size, 1.0 X digital zoom, pinhole 1.0 AU (GluA1 was visualized with 2.0 AU) with a line average of 2.0 using a 63× oil-immersion objective (1.4 NA). Astrocytes were imaged if the entirety of the cell and astrocytic arbors could be visualized with low background noise (Siemsen et al., 2023).

### 2.7. Image analysis

Following confocal image acquisition, Z-stack images were exported to Bitplane Imaris v. 9.1 (Oxford Instruments, Concord, MA) for rendering and subsequent analysis according to methods previously published (Siemsen et al., 2023). Once each individually labeled astrocyte cell was isolated, a 3D surface was rendered from the LCK-GFP or PMCA signal. Then, the LCK-GFP or PMCA signal was isolated to each individual astrocyte render to remove the background. Using the space filling rendering of the virally labeled astrocyte, the number of GluA1 puncta were quantified within the territory of the virally labeled cell. Astrocytic interaction with synapses was assessed by measuring colocalization of the astrocyte plasma membrane signal (LCK-GFP or PMCA-mCherry) with the synaptic marker GluA1. This signal was also rendered a same space filling model and summed. After dividing this summed astrocyte interaction volume by the number of GluA1 puncta within the territory of the virally labeled astrocyte, a per synapse astrocyte interaction score was generated for each cell. This interaction score reflects the degree of astrocytic ensheathment of GluA1 labeled synapses. Thresholds were set empirically by investigators blind to treatment conditions. Any images that contained uneven signal intensity distribution throughout the image were not analyzed and the voxel values above the signal intensity threshold were considered colocalized with a higher value indicating a higher degree of astrocyte synaptic GluA1 interaction. The data collected included the ratio of the astrocyte surface area to the volume, the number of GluA1 puncta per astrocyte normalized to astrocyte cell volume, and the degree of colocalization determined by an IMARIS derived sum of synaptic interaction normalized to the number of GluA1 puncta per astrocyte.

### 2.8. Two-bottle choice limited access drinking

Following viral infusion surgery, mice were given 2 weeks to recover in their homecage before undergoing 4 weeks of baseline drinking using a two-bottle choice limited access model. On each day (Monday-Friday), mice were given bottles containing either tap water or 15% ethanol 3 h into the dark cycle and allowed to drink for 2 h. Bottle positions were alternated each day to avoid side preference and were weighed before and after the drinking session to determine the amount of alcohol and water consumed. To account for volume lost during bottle movement, two spill cages were utilized as controls. Following 4 weeks of baseline drinking, mice were counterbalanced into CIE or Air groups based on their baseline alcohol consumption and then exposed to four cycles of CIE or Air exposure each interleaved with a test week of homecage drinking.

### 2.9. Behavioral battery paradigm

Virus infused male mice (LCK-GFP N = 6, PMCA N = 6) underwent habituation and handling for an hour in the testing environment 3 days prior to the first test day. Mice first underwent locomotor testing by placing them in a clean homecage with a single layer of bedding surrounded by an infrared beam array (San Diego Instruments, Photobeam Activity System). Locomotor behavior was quantified as beam breaks over a 1-h period broken into 5-min bins. The following day, mice were placed in a well-lit (~120 lux center) open field arena (44 cm^2^) for 5 min and their activity was tracked by a video camera using ANY-Maze software (Stoelting Co, Wood Dale, IL). Mice were then tested for anxiety-like behavior using an elevated plus maze (Stoelting #60140) illuminated with white light (~120 lux). ANY-maze software was used to quantify distance traveled and time spent in open or closed arms during the 5-min test period. The next day, spontaneous alternation was tested using a Y-maze (Stoelting #60180) illuminated with dim white light (~30 lux). Correct alternations were defined by the number of times a mouse entered a series of 3 different arms without re-entering a previously explored arm. Lastly, mice were tested for spatial learning using the Barnes Maze. On the initial trial, mice were acclimated to the escape chamber for 2 min before testing. During testing, mice were introduced to the center of the Barnes Maze (Stoelting #60170) in bright white light (~250 lux) with 4 distinct spatial cues evenly distributed around the arena. Mice were allowed to freely explore the arena for 5 min or until they entered the escape chamber. Latency to escape was recorded for each trial and mice failing to find the escape chamber at the end of the trial were guided to the escape hole. At the end of each trial whether the mouse was guided to the hole or not, they were allowed to rest in the escape chamber for 2 min. Each mouse was tested twice a day, and data reflects the average latency to enter the escape hole over the two trials per day. Following training, a 72-h short-term recall, 1-month long-term recall, and reversal learning tests were conducted.

### 2.10. Statistical analysis

Current evoked spiking was analyzed with Prism 10 Software (GraphPad Inc. San Diego, CA) using a two-way repeated measures ANOVA or mixed effect analysis with Šidák’s multiple comparisons test where appropriate. Action potential characteristics were analyzed between treatment group (Air or CIE) and viral group (LCK-GFP or PMCA) using a two-way ANOVA Holm-Šidák’s when necessary. Confocal images were analyzed using two-way ANOVA (GraphPad Inc. San Diego, CA) and all measures were normalized to cell volume to control for any differences in cell size within groups. When appropriate, a Holm-Šidák’s multiple comparisons test post-hoc analysis was utilized. For the two-bottle choice drinking data, the amount of alcohol consumed during a session was reported in g/kg and data was analyzed using a two-way ANOVA and Holm-Šidák’s multiple comparisons test as necessary. Lastly, the behavioral battery assessment was analyzed using an unpaired *t*-test or two-way ANOVA with Holm-Šidák’s multiple comparisons test used to assess differences between viral groups when necessary. In all statistical tests, a p < 0.050 was considered statistically significant.

## 3. Results

### 3.1. Effects of CIE and astrocyte PMCA on OFC neuron excitability

**Fig. 1.**
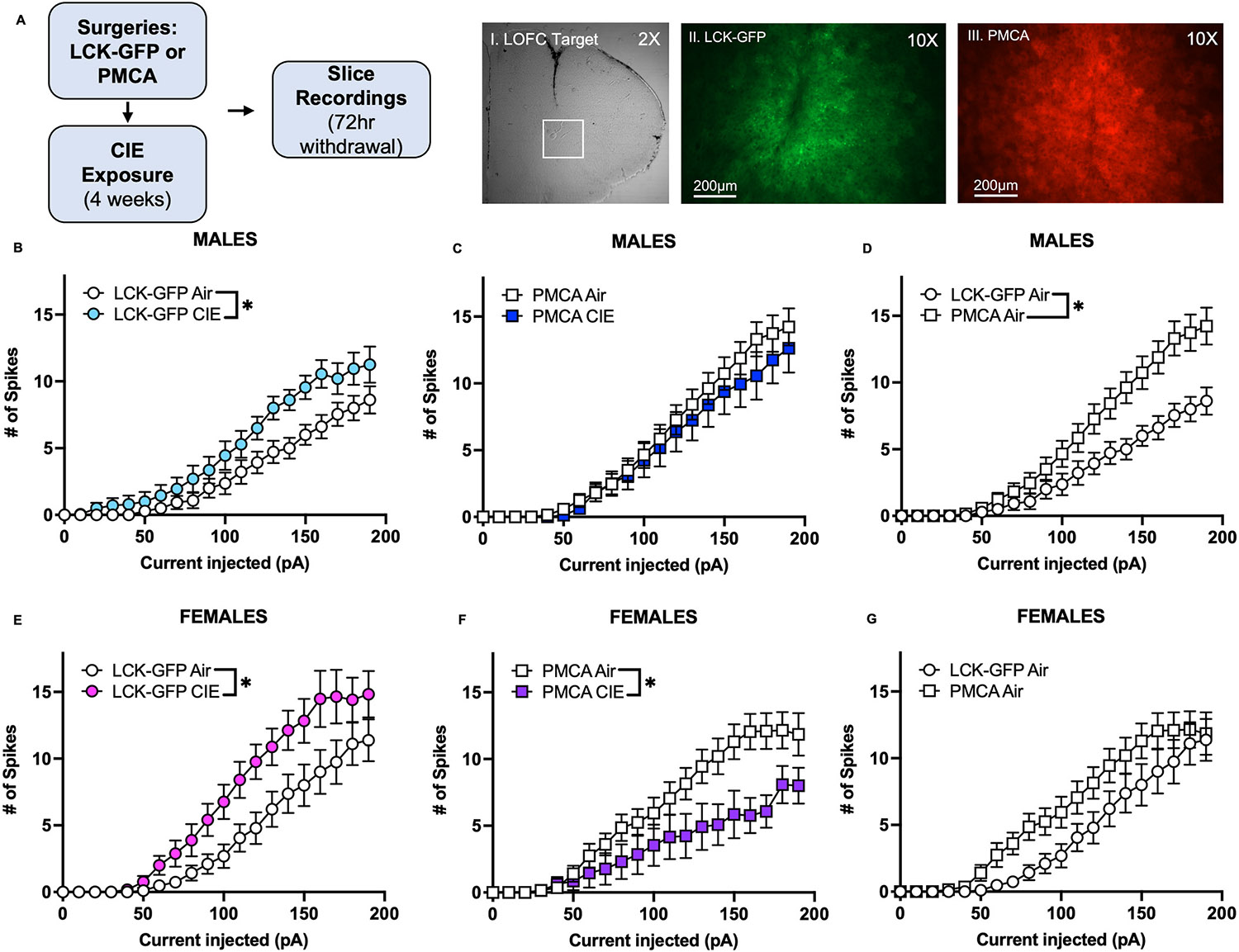
Effects of CIE and Astrocyte PMCA on OFC Neuron Excitability. (**A**) Schematic shows timing of viral surgeries, CIE exposure, and slice electrophysiology recordings. Representative images show I. OFC virus target, II. LCK-GFP (green), and III. PMCA (red) in OFC astrocytes. Scale bar: 2X and 10X (200 μm). Graphs (**B–G**) show the number of spikes generated by OFC neurons from Air or CIE treated mice over the range of injected current steps (0–190; 750msec each). Data are presented as mean ± SEM. (**B**) CIE exposure significantly increased the number of action potentials of OFC neurons in male LCK-GFP CIE mice as compared to Air exposed mice. (**C**) OFC neuron spiking was not different between Air and CIE treated PMCA male mice. (**D**) PMCA expression alone increased OFC neuron firing in Air treated male mice as compared to LCK-GFP Air mice. (**E**) CIE exposure significantly increased spike firing of OFC pyramidal neurons of female LCK-GFP mice compared to Air controls. (**F**) Spike firing in CIE treated female mice was reduced compared to Air controls. (**G**) There was a non-significant trend towards an increase in firing of Air treated female PMCA mice as compared to LCK-GFP Air controls. Symbol (*): p < 0.05.

Following infusion of astrocyte-specific LCK-GFP or PMCA AAVs in the OFC, male and female mice underwent 4 consecutive weeks of CIE exposure and slice electrophysiology recordings were performed 72 h following the last CIE cycle (Fig. 1A). CIE exposure significantly increased current-evoked firing of OFC pyramidal neurons in LCK-GFP male mice as compared to Air controls (mixed-effects analysis, main effect of CIE: F_(1,32)_ = 6.36, p = 0.017, Air N = 14 neurons/6 mice; CIE N = 20 neurons/7 mice, Fig. 1B). In contrast, action potential firing across the current steps was not different between Air and CIE treated PMCA male mice (two-way ANOVA: F_(1,40)_ = 0.48, p = 0.49, Air N = 26 neurons/6 mice, CIE N = 16 neurons/5 mice, Fig. 1C). Closer inspection of the data revealed that PMCA itself enhanced firing in Air treated male mice (mixed effect analysis, main effect of PMCA: F_(1,38)_ = 6.21, p = 0.017, Fig. 1D). CIE exposure also significantly increased the excitability of OFC neurons in female LCK-GFP mice (two-way ANOVA, main effect of CIE: F_(1,34)_ = 5.38, p = 0.027, Air N = 19 neurons/6 mice, CIE N = 17 neurons/6 mice, Fig. 1E). In contrast to male mice, CIE exposure significantly reduced spiking in PMCA expressing female mice below that of Air controls (two-way ANOVA, main effect of CIE: F_(1,31)_ = 5.56, p = 0.025, Air N = 20 neurons/5 mice; CIE N = 13 neurons/5 mice, Fig. 1F). PMCA expression itself also appeared to increase current evoked spiking in Air exposed female mice but this did not quite meet statistical significance (two-way ANOVA: F_(1,37)_ = 3.84, p = 0.058, Fig. 1G).

Action potential kinetics, rheobase, input resistance, and membrane capacitance of the electrophysiological recordings were analyzed to further investigate the effects of PMCA and CIE exposure on spiking. In male mice, the membrane capacitance of PMCA Air mice was significantly less than that of male LCK-GFP Air mice (two-way ANOVA, main effect of Virus: F_(1,72)_ = 5.72, p = 0.019; post-hoc Holm-Šidák’s multiple comparisons test: LCK-GFP Air vs. PMCA Air t = 3.34, p = 0.0027, Table 1). There was also a Treatment × Virus interaction for cell capacitance (two-way ANOVA: F_(1,72)_ = 5.28, p = 0.025). The membrane capacitance of LCK-GFP CIE male mice was lower compared to Air controls but did not reach statistical significance (post-hoc Holm-Šidák’s multiple comparisons test: LCK-GFP Air vs. LCK-GFP CIE t = 2.18, p = 0.064, Table 1). In female mice, there was a Treatment × Virus Interaction for rheobase (two-way ANOVA: F_(1,65)_ = 10.81, p = 0.0016, Table 1). CIE exposure increased the rheobase of PMCA females compared to Air controls (post-hoc Holm-Šidák’s multiple comparisons test: PMCA Air vs. PMCA CIE t = 2.56, p = 0.026, Table 1). In contrast, the rheobase of CIE exposed LCK-GFP female mice was decreased compared to Air controls (post-hoc Holm-Šidák’s multiple comparisons test: LCK-GFP Air vs. LCK-GFP CIE t = 2.08, p = 0.042, Table 1). CIE exposed female PMCA mice also had a higher rheobase as compared to LCK-GFP CIE female mice (post-hoc Holm-Šidák’s multiple comparisons test: LCK-GFP CIE vs. PMCA CIE t = 2.72, p = 0.017, Table 1). Lastly, there was a main effect of treatment (two-way ANOVA, main effect of CIE: F_(1,65)_ = 7.89, p = 0.0065, Table 1) and a Treatment × Virus interaction for input resistance in female mice (two-way ANOVA: F_(1,65)_ = 4.84, p = 0.031, Table 1). Post-hoc testing revealed a significant difference in the input resistance between Air and CIE treated PMCA mice (post-hoc Holm-Šidák’s multiple comparisons test: PMCA Air vs. PMCA CIE t = 3.43, p = 0.0021, Table 1).

### 3.2. Effects of CIE and PMCA on OFC Astrocyte Morphology and Astrocyte synaptic interaction

**Fig. 2.**
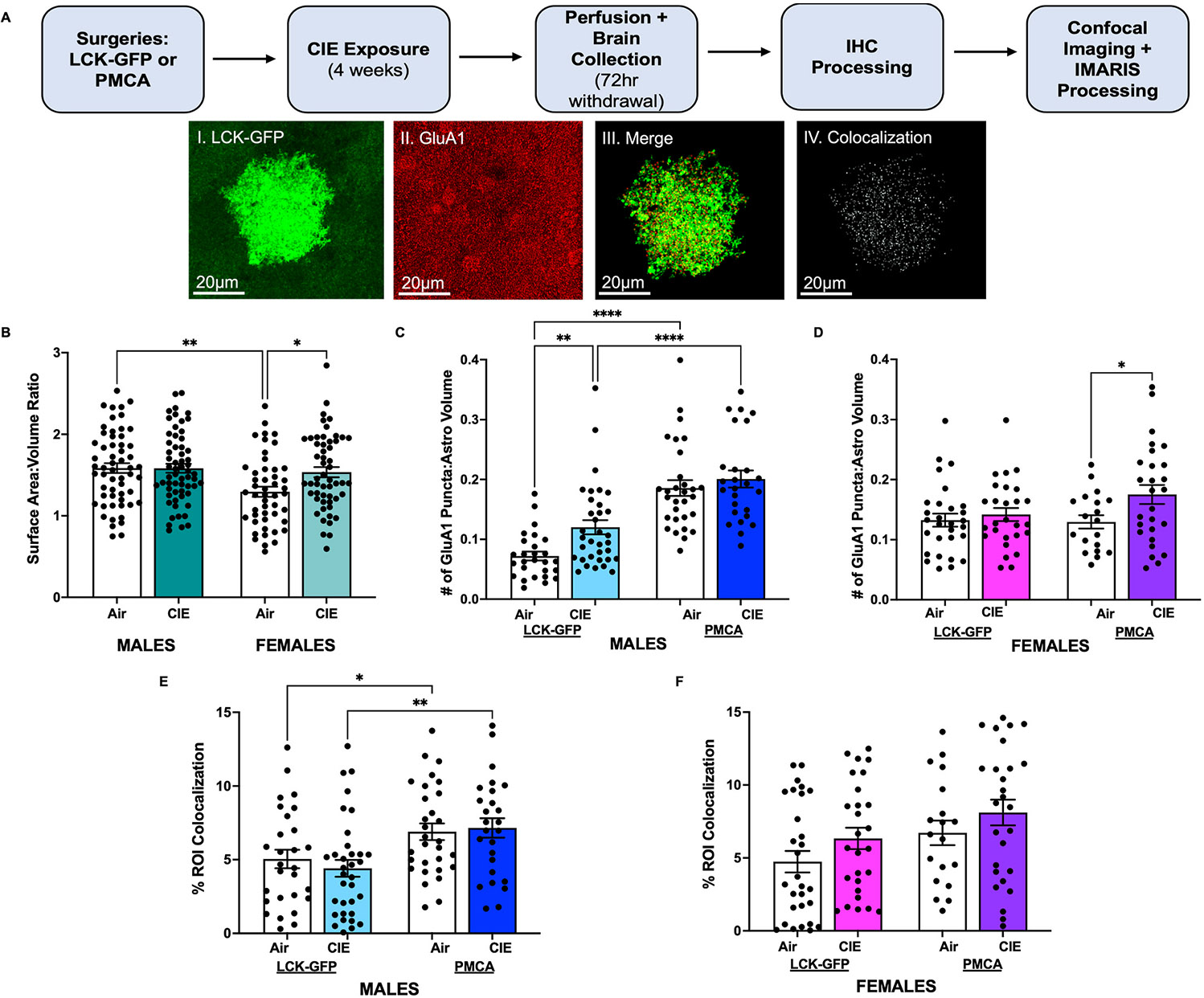
Effects of CIE and PMCA on OFC Astrocyte Morphology and Astrocyte-Neuron Interactions (**A**) Schematic shows timeline of viral surgeries, CIE exposure, and confocal imaging. Representative confocal images show a single OFC astrocyte with expression of LCK-GFP (green), GluA1 puncta (red), merged LCK-GFP/GluA1 images, and colocalization of the astrocyte with GluA1 puncta (scale bars; 20 μm). Data are presented as mean ± SEM. (**B**) Astrocyte surface area:volume ratio of Air and CIE treated male and female mice. Astrocytes from Air treated female mice were smaller compared to those from Air treated male mice and CIE exposure increased the surface area:volume ratio of astrocytes in female mice. (**C**) CIE exposure increased the number of GluA1 puncta within the vicinity of the astrocyte volume in LCK-GFP male mice. PMCA expression alone increased the number of GluA1 puncta in both Air and CIE treated male mice. (**D**) There was no effect of CIE exposure on the number of GluA1 puncta per astrocyte volume in female LCK-GFP mice. However, CIE exposure did increase the number of GluA1 puncta per astrocyte volume in PMCA female mice. (**E**) PMCA increased the colocalization of GluA1 and OFC astrocytes in both male Air and CIE exposed mice. (**F**) There was a difference in the colocalization of GluA1 puncta and astrocytes between LCK-GFP and PMCA female mice. CIE exposure did not affect the colocalization of GluA1 puncta and astrocytes in either LCK-GFP or PMCA exposed females. Symbols (*), (**), (****): p < 0.05, p < 0.01, or p < 0.0001, respectively.

To complement the electrophysiology studies, confocal microscopy was used to determine the effects of PMCA and CIE exposure on OFC astrocytes and their interaction with excitatory glutamatergic synapses in Air and CIE treated mice. Fig. 2A shows representative confocal images (GluA1, LCK-GFP, merge, and colocalization) of a single OFC astrocyte following IMARIS image processing. For all groups, 4–6 astrocytes per animal (males: N = 6 mice, females N = 4–6 mice) were imaged for both viral (LCK-GFP or PMCA) and treatment (Air or CIE) groups. The initial analysis assessed the effects of CIE exposure on astrocyte size measured as the ratio of the astrocyte surface area to the volume. Group means (±SEM) for each condition were: Males: LCK-GFP Air: 1.66 ± 0.075; PMCA Air: 1.52 ± 0.092; LCK-GFP CIE: 1.56 ± 0.079; PMCA CIE: 1.61 ± 0.077 and Females: LCK-GFP Air: 1.29 ± 0.076; PMCA Air: 1.30 ± 0.11; LCK-GFP CIE: 1.39 ± 0.074; PMCA CIE: 1.68 ± 0.095. Analysis of this data revealed that astrocyte area/volume of Air and CIE groups were similar within each sex regardless of virus (three-way ANOVA (Air vs. CIE) x (LCK-GFP vs. PMCA) x (Males vs. Females): F_(1,211)_ = 0.20, p = 0.66) and so the viral groups were combined. At baseline, astrocytes from female Air mice had a smaller surface area/volume ratio as compared to male mice (two-way ANOVA, main effect of Sex: F_(1,215)_ = 7.85, p = 0.0055; post-hoc holm-Šidák’s multiple comparisons test: Male Air vs. Female Air t = 3.34, p = 0.002, Fig. 2B). There was also a Sex × CIE interaction in female mice (two-way ANOVA: F_(1,215)_ = 4.09, p = 0.045; post-hoc Sidák’s multiple comparisons test: Female Air vs. Female CIE t = 2.73, p = 0.014).

CIE exposure increased the number of GluA1 puncta within the territory of virally labeled astrocytes in males of both viral groups (two-way ANOVA, main effect of CIE: F_(1, 113)_ = 6.85, p = 0.01; post-hoc Holm-Sidák’s multiple comparisons test: LCK-GFP Air vs. LCK-GFP CIE t = 2.87, p = 0.0098, Fig. 2C). PMCA expression alone increased the number of puncta within the territory of virally labeled astrocytes in both Air and CIE males (two-way ANOVA, main effect of Virus: F_(1, 113)_ = 64.35, p < 0.0001; post-hoc Holm-Šidák’s multiple comparisons test: LCK-GFP Air vs. PMCA Air t = 6.58, p < 0.0001; LCK-GFP CIE vs. PMCA CIE t = 4.75, p < 0.0001, Fig. 2C). CIE exposure did not affect the number of GluA1 puncta within the territory of LCK-GFP labeled astrocytes in female mice but increased puncta number in PMCA females (two-way ANOVA, main effect of CIE: F_(1, 98)_ = 4.62, p = 0.034; post-hoc Holm-Sidák’s multiple comparisons test: PMCA Air vs. CIE t = 2.39, p = 0.037, Fig. 2D). In males, CIE exposure did not increase the physical interaction of astrocytes with GluA1 puncta, but PMCA increased astrocyte-synapse interaction in both Air and CIE exposed mice (two-way ANOVA, main effect of virus: F_(1, 114)_ = 14.36, p = 0.0002; post-hoc Holm-Sidák’s multiple comparisons test: LCK-GFP CIE vs. PMCA CIE t = 3.21, p = 0.0034; LCK-GFP Air vs. PMCA Air t = 2.15, p = 0.034, Fig. 2E). PMCA expression also impacted astrocyte-synapse contact in females (two-way ANOVA, main effect of virus: F_(1, 98)_ = 5.32, p = 0.023, Fig. 2F), but there were no significant post-hoc comparisons. Interestingly, there was a trend for increased astrocyte-synapse interaction following CIE exposure in both the LCK-GFP and PMCA groups of female mice, but this did not reach statistical significance (two-way ANOVA, main effect of CIE: F_(1, 98)_ = 3.38, p = 0.069, Fig. 2F).

### 3.3. Effects of CIE and astrocyte PMCA on alcohol consumption

**Fig. 3.**
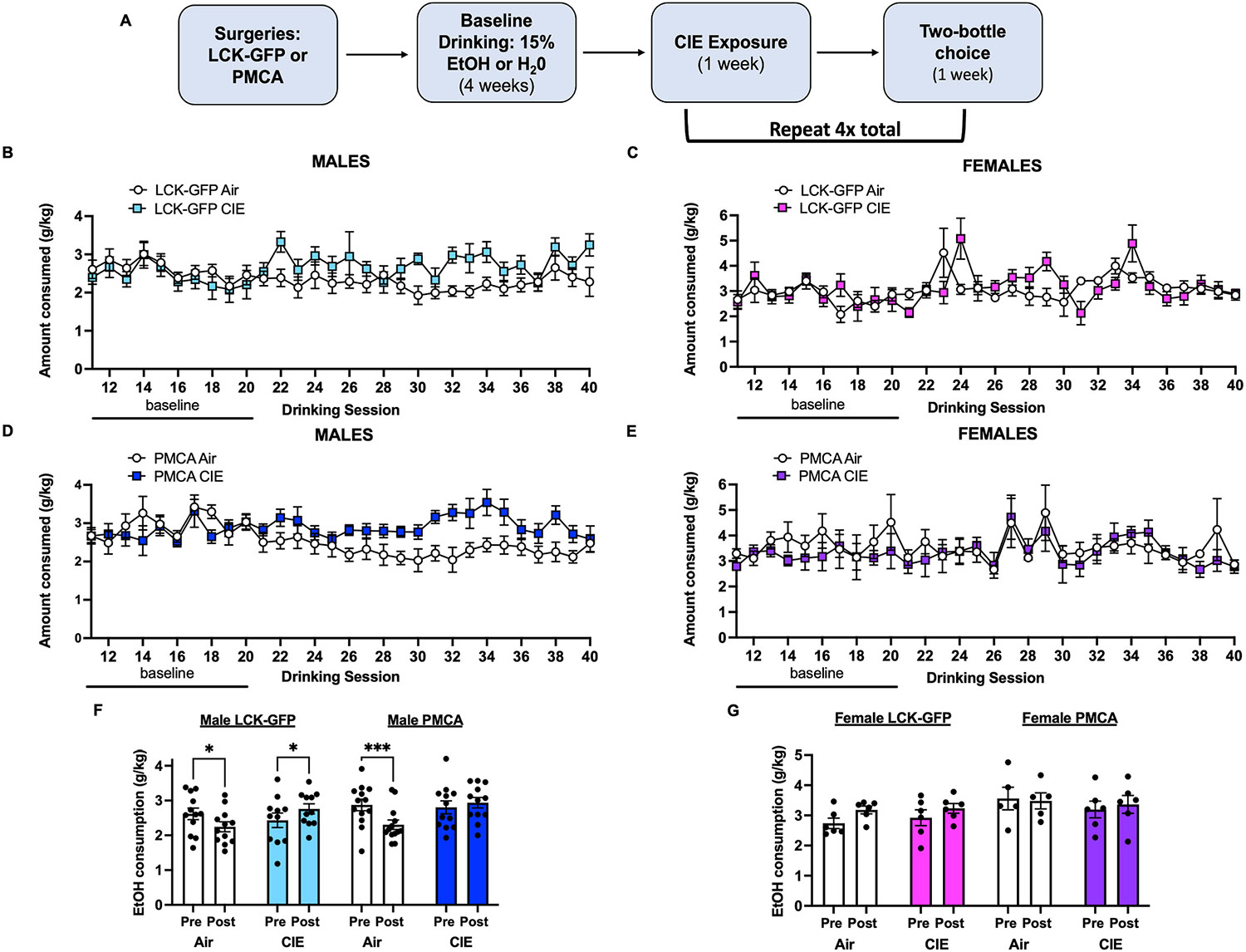
Effects of CIE and Astrocyte PMCA on Alcohol Consumption. (**A**) Schematic shows the timeline of viral surgeries, CIE or Air exposures, and interleaved drinking sessions. All data are presented as mean ± SEM. Average daily g/kg alcohol consumed by LCK-GFP and PMCA male (**B**, **D**) and female (**C**, **E**) mice for the last 2 weeks of baseline and four weeks of post-CIE or Air drinking. The average of the last two weeks of baseline drinking is defined as “Pre” and the average of the four weekly cycles of drinking after either Air or CIE exposure is defined as “Post”. (**F**) CIE exposure had a main effect on alcohol drinking that was driven by an increase in alcohol consumption of LCK-GFP but not PMCA CIE exposed male mice. Both male Air LCK-GFP and PMCA mice showed decreased alcohol consumption over the four weeks of post-baseline drinking. (**G**) Neither LCK-GFP nor PMCA female mice exhibited changes in alcohol drinking before or after Air or CIE. Symbols (*) or (***): p < 0.05 or p < 0.001, respectively.

Male and female mice infused with an astrocyte-specific LCK-GFP or PMCA virus in the OFC underwent four consecutive weeks (Monday-Friday) of two bottle-choice drinking of water or 15% ethanol. Mice were then counterbalanced into CIE (LCK-GFP: males N = 11, females N = 6; PMCA: males N = 12, females N = 6) or Air (LCK-GFP: males N = 12, females N = 6; PMCA: males N = 13, females N = 5) groups based on alcohol consumption during baseline drinking sessions. They were then exposed to four weekly cycles of CIE or Air exposure each interleaved with a test week of homecage drinking (Fig. 3A). To compare changes in alcohol consumption between baseline and treatment within each animal, the amount of alcohol consumed by each mouse was calculated as the average of the last two weeks of baseline drinking (“Pre”) and the average of the four weekly cycles of drinking after either Air or CIE exposure (“Post”). There was a significant effect of Treatment on the amount of alcohol consumed (two-way ANOVA, main effect of CIE: F_(3,44)_ = 8.11, p = 0.0002, Fig. 3F), and further analysis revealed that this was driven by an increase in drinking following CIE exposure in LCK-GFP expressing male mice (post-hoc analysis Holm-Šidák’s multiple comparisons test: LCK-GFP CIE Pre vs. Post t = 2.13, p = 0.039, Fig. 3F). In contrast, CIE exposure did not increase alcohol consumption in PMCA mice (post-hoc analysis Holm-Šidák’s multiple comparisons test: PMCA CIE Pre vs. Post t = 0.91, p = 0.37, Fig. 3F). Interestingly, in both groups of Air exposed male mice, alcohol consumption significantly declined over the four weeks of post-baseline drinking (post-hoc analysis Holm-Šidák’s multiple comparisons test: LCK-GFP Air Pre vs. Post t = 2.53, p = 0.015; PMCA Air Pre vs. Post t = 4.012, p = 0.0002, Fig. 3F). Unlike male mice, there were no virus (two-way ANOVA, LCK-GFP or PMCA: F_(3,19)_ = 1.32, p = 0.3, Fig. 3G) or treatment (two-way ANOVA, Air or CIE: F_(1,19)_ = 2.44, p = 0.13, Fig. 3G) effects on alcohol drinking in female mice.

### 3.4. Effects of astrocyte PMCA on Standard Behavioral Measures

**Fig. 4.**
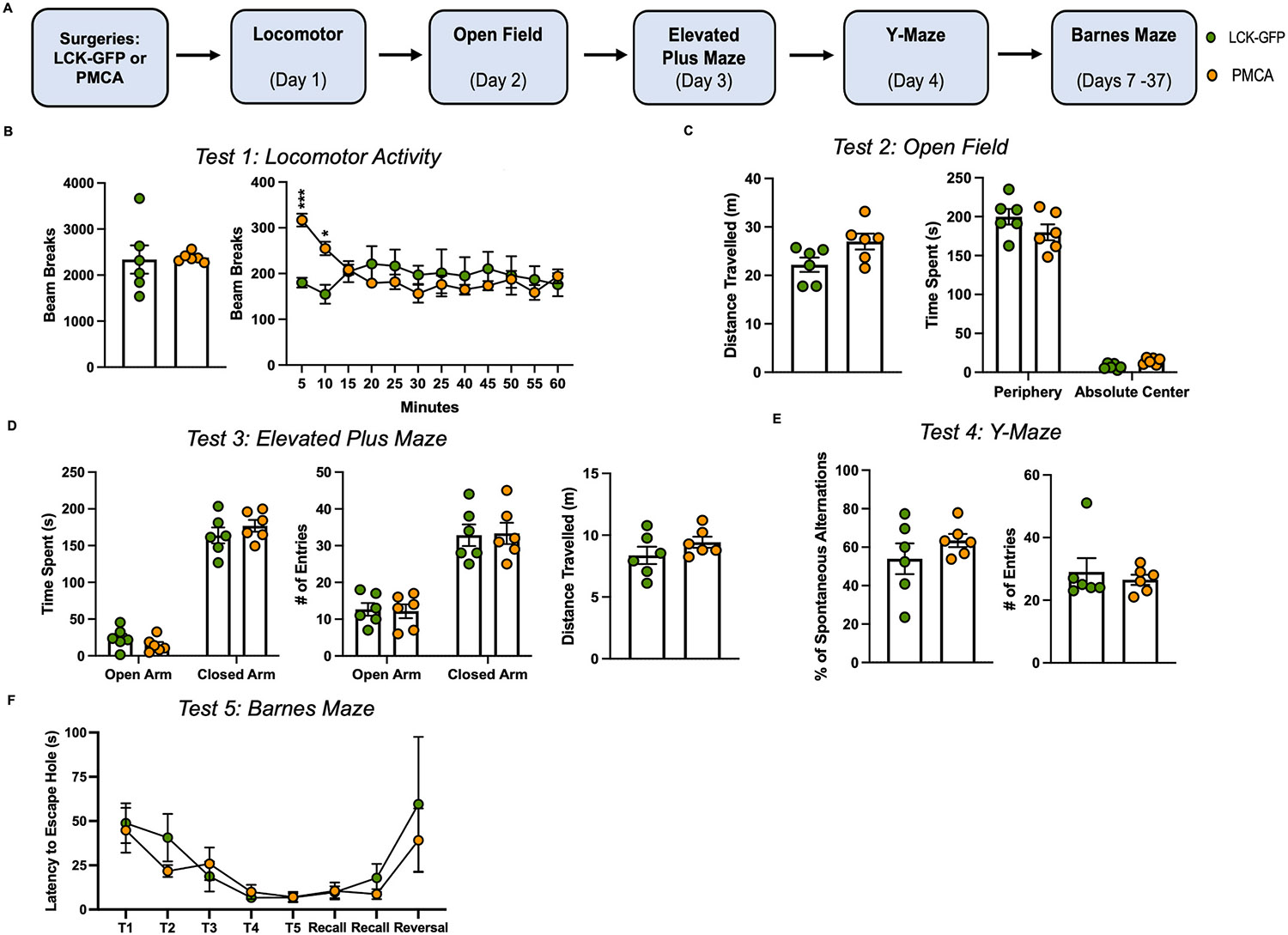
Effect of Astrocyte PMCA on Standard Behavioral Measures. (**A**) Schematic showing timeline of viral surgeries and the behavioral test battery. All data are presented as mean ± SEM. (**B**) Locomotor activity: PMCA males were significantly more active during the first 10 min of the test. There were no differences in behavior between LCK-GFP and PMCA mice following testing in: (**C**) Open Field (total distance, time spent in periphery or center), (**D**) Elevated Plus Maze (time spent or entries into open or closed arm), (**E**) Y-Maze (percent spontaneous alternation), or (**F**) Barnes Maze (latency to escape, short and long-term memory recall). Symbols (*) or (***): p < 0.05 or p < 0.001, respectively.

Separate cohorts of male PMCA and LCK-GFP mice underwent a battery of behavioral tests to assess whether expression of PMCA in OFC astrocytes alters basic motor and exploratory behaviors (Fig. 4A). There was no difference in the total number of beam breaks between LCK-GFP and PMCA mice during the total locomotor box session. However, when the session was broken down into 5-min bins, there was a Time × Virus interaction where PMCA mice exhibited significantly higher locomotor activity compared to LCK-GFP mice during the first 10 min of the session (two-way ANOVA: F_(11,110)_ = 4.87, p < 0.0001; post-hoc Holm-Šidák’s multiple comparisons test: 5-min LCK-GFP vs. PMCA p = 0.0003; 10-min LCK-GFP vs. PMCA p = 0.037, Fig. 4B). The distance traveled during the Open Field test was slightly increased in PMCA mice compared to controls although this did not reach statistical significance (Unpaired *t*-test: t_(10)_ = 2.16, p = 0.056, Fig. 4C). There were no significant differences in behavior between LCK-GFP and PMCA mice in other aspects of the Open Field test (time spent in periphery or absolute center), or in Elevated Plus Maze (time spent or entries into open or closed arm), Y-Maze (percent spontaneous alternation), or Barnes Maze (latency to escape, short and long-term memory recall; Fig. 4C-F).

## 4. Discussion

In this study, we examined the role of OFC astrocytes in mediating physiological, structural, and behavioral alterations that follow chronic ethanol exposure. This was tested by expressing the membrane-bound calcium exporter PMCA selectively in OFC astrocytes to blunt astrocytic calcium signaling. The results suggest that there are sex-dependent differences in how OFC astrocytes and neurons interact and respond to chronic ethanol. For example, CIE exposure increased neuronal firing in control male and female mice while PMCA itself increased firing in male mice but prevented the CIE-induced increase in firing in female mice. Similarly, both PMCA and CIE exposure increased the number of GluA1 containing synapses within astrocyte territories in male but not female mice while PMCA alone increased astrocytic interaction with excitatory synapses in a similar sex-dependent manner. CIE exposure enhanced alcohol consumption in male but not female mice and this increase was prevented by PMCA expression. The PMCA-induced changes in neuronal excitability and astrocyte-neuron interaction observed in this study occurred in the absence of significant changes in behavior.

### 4.1. PMCA, neuronal physiology and astrocyte morphology

The present study is among the first to describe how chronic alcohol exposure affects astrocytes and their interaction with neurons in the OFC. Importantly, it sheds light on the possible mechanisms underlying the CIE-induced increases in current evoked spiking in this neuronal population (Nimitvilai et al., 2016; Nimitvilai-Roberts et al., 2021). The PMCA construct used in the present study has been previously shown to reduce intracellular astrocytic calcium signaling involved with downstream calcium-dependent processes such as gliotransmitter release (Yu et al., 2018). As our previous study showed that expressing PMCA in OFC astrocytes reduced the inhibition of OFC neurons following an acute ethanol wash (Nimitvilai-Roberts et al., 2021), we initially hypothesized that it would prevent or reduce the hyperexcitabilty of OFC neurons observed following withdrawal from CIE exposure. Surprisingly, in male mice, PMCA expression itself induced changes in neuronal physiology and astrocyte morphology that were similar to those following CIE exposure alone. Currently, the mechanism(s) underlying the effect of PMCA on OFC neuronal firing and GluA1-OFC astrocyte interaction remains unknown. While the findings from the present study support the hypothesis that astrocytes are involved in mediating the physiological responses to chronic alcohol exposure, blunting astrocytic calcium signaling may have engaged a myriad of cellular adaptations linked to both astrocytic and neuronal function. As an example, gliotransmitters such as ATP and glutamate can be released in a calcium-independent pathway (Xiong et al., 2018; Neary et al., 1999) and the extent of which these pathways occur is environmentally dependent and can be triggered by stress or inflammation (Xiong et al., 2018). Chronic alcohol exposure has been shown to increase levels of stress and inflammation in both clinical and preclinical models (Crews et al., 2017; Kelley and Dantzer, 2011; Flores-Bastias and Karahanian, 2018; Wang et al., 2010; Lowe et al., 2020). Furthermore, chronic stress has been shown to alter astrocyte morphology in the prefrontal cortex of male mice (Codeluppi et al., 2021). Therefore, the ability of PMCA to induce CIE-like increases in OFC excitability, GluA1 expression, and an overall increase in synaptic colocalization may be driven by astrocytic release of gliotransmitters.

To meet the needs of the cell following inflammation and damage, different astrocyte receptors can be upregulated to promote ATP release through a calcium-independent mechanism (Neary et al., 1999). The released ATP is then converted into adenosine that can bind to and activate inhibitory adenosine receptors. In the frontal cortex, this promotes a homeostatic increase in glutamatergic neuron excitability (Kerkhofs et al., 2018). Ethanol exposure has also been shown to increase adenosine levels and ultimately raise extracellular cAMP signaling cascades leading to the translocation of PKA (Nagy et al., 1989; Asher et al., 2002; Gordon et al., 1986; Sapru et al., 1994). In a previous study, an adenosine 2A receptor antagonist blocked the ethanol-induced translocation of PKA in rat hippocampal primary culture cells (Yao et al., 2002). Extracellular adenosine levels are regulated by the equilibrative nucleoside transporter 1 (ENT1) and male mice lacking this transporter show escalated alcohol consumption during a two-bottle choice paradigm that is reversed following viral infusion of ENT1 into the nucleus accumbens (Jia et al., 2020). As astrocyte PMCA expression in male mice induced hyperexcitability of OFC neurons similar to that observed following CIE exposure, this finding in conjunction with the previously reported studies suggests that as homeostatic calcium-dependent pathways are disrupted, calcium-independent mechanisms may play a larger role in modulating astrocyte synaptic interactions and regulating neuronal excitability.

### 4.2. Regional specificity and sex-dependent differences in astrocytes following chronic drug exposure

There is regional specificity regarding the effects of alcohol on astrocyte morphology and density. In clinical populations, individuals with AUD show reduced astrocytic density in the orbitofrontal and prefrontal cortex and hippocampus (Miguel-Hidalgo et al., 2006). Reduced prefrontal cortex astrocyte density is also observed in preclinical models of high alcohol preferring rats prior to alcohol exposure (Miguel-Hidalgo, 2005). However, changes in astrocytic density are also dependent on the alcohol model and brain region of interest. As an example, male rats have increased astrocyte density in cortical regions following repeated doses of alcohol or after consuming an alcohol-rich diet (Udomuksorn et al., 2011; Dalcik et al., 2009). Astrocyte density is also increased in the prelimbic sub-division of the medial prefrontal cortex (mPFC) of alcohol preferring rats during alcohol abstinence (Miguel-Hidalgo, 2006). Furthermore, the density of nucleus accumbens (NAc) core astrocytes in male rats is increased following alcohol self-administration, with no change in astrocytic density noted in the NAc shell (Bull et al., 2014). The duration of exposure is also an important variable in determining the impact of alcohol on astrocytes. Following every other day intermittent alcohol exposure, astrocytic density is reduced in the prelimbic and anterior cingulate cortex whereas astrocytic density in the infralimbic and orbitofrontal cortex remains unchanged (Bull et al., 2015). Interestingly, following 3 weeks of abstinence after continuous homecage alcohol drinking, there was a reduction in prelimbic mPFC and OFC astrocyte density, with similar results observed in the OFC following operant alcohol self-administration (Bull et al., 2015).

In the present study, OFC astrocytes in ethanol naive female mice had a smaller basal surface area to volume ratios. Previous studies show that OFC neurons in female mice are less sensitive to acute ethanol inhibition than those in male mice (Nimitvilai et al., 2020). The decreased morphometric features of female OFC astrocytes shown in this current study could contribute to this effect as a smaller astrocytic area/volume may reduce the ability of glycine released from astrocytes to interact with inhibitory glycine receptors on OFC neurons (Nimitvilai-Roberts et al., 2021). However, the present study also showed that CIE increased the area/volume ratio of OFC astrocytes in female mice. This wouldn’t explain the reduced sensitivity of OFC neurons in female mice to acute ethanol following CIE exposure (Nimitvilai et al., 2020). While there are fewer studies that have assessed the impact of alcohol on astrocytes in females compared to males, findings from recent reports highlight the sexually dimorphic nature of astrocytes in adapting to alcohol exposure. For example, female mice showed increased astrocytic density in the hippocampus compared to males (Wilhelm et al., 2016) while GFAP immunoreactivity was reduced in the NAc of female mice (Giacometti et al., 2020). The present study provides additional evidence for sex-dependent differences in astrocytes as the expression of PMCA alone increased the colocalization of OFC astrocytes and GluA1 puncta in male but not female mice.

Similar to ethanol, other drugs of abuse produce sex-dependent effects on astrocyte morphology and synaptic interaction and these actions are contingent on brain region and duration of drug exposure. For example, administration of D-amphetamine in male rats increased GFAP expression in the hippocampus and prefrontal cortex (Permpoonputtana et al., 2012) and chronic methamphetamine treatment increased GFAP expression and astrocyte size in the hippocampus of female mice (Castellano et al., 2016). In addition, following abstinence from long-term self-administration of cocaine, male but not female rats showed altered astrocyte morphology and decreased synaptic colocalization to PSD-95 in the NAc (Kim et al., 2022). A similar finding was observed after cocaine self-administration and extinction as GFAP expression and colocalization of astrocytes with synapsin I were reduced in the NAc core of male rats, yet female rats were not investigated (Scofield et al., 2016). In contrast, following heroin self-administration and extinction, GFAP expression in male rats was increased in astrocytes surrounding prelimbic mPFC neurons that project to the nucleus accumbens along with increased astrocyte co-localization to GluA2 and synapsin (Siemsen et al., 2023).

The current study used the calcium exporter PMCA to blunt intracellular calcium signaling and reduce astrocyte activity (Yu et al., 2018). In contrast, previous studies from our group and others have examined drug seeking behavior in animals following chemogenetic activation of Gq-signaling in astrocytes, that results in elevations in intracellular calcium. Activating astrocytes in the NAc reduced reinstatement of cue-induced cocaine seeking by restoring tone on glutamatergic presynaptic metabotropic glutamate receptor 2/3 and preventing cocaine-induced glutamate overflow (Scofield, 2018; Scofield et al., 2015). Chemogenetic activation of astrocytes in the NAc core reduced motivation for alcohol seeking following 3 weeks of abstinence (Bull et al., 2014). CIE exposure blocked the extinction of food-associated conditioned place preference in female mice and this was restored by activating Gq-DREADDs expressed in NAc astrocytes (Giacometti et al., 2020) Taken together, these findings and others suggest that astrocytic calcium signaling plays a critical role in adaptations following chronic drug use, such as alcohol, and it’s modulatory effects is heavily influenced by drug type, duration of exposure, brain region, and sex.

### 4.3. PMCA and alcohol consumption following CIE

In the present study, CIE exposed male but not female mice increased their alcohol consumption and this was prevented by expressing PMCA in OFC astrocytes. The OFC’s ability to regulate alcohol drinking appears to require maintaining optimal neuronal activity in specific OFC subregions. For example, chemogenetic inhibition of lateral OFC neurons increased alcohol consumption in CIE treated mice (den Hartog et al., 2016) while inhibiting medial OFC neurons had no effect on drinking in non-dependent mice using a limited access paradigm (Schuh et al., 2022). In the mPFC, activating astrocytes via G_q_-DREADDs increased alcohol consumption during the initial sessions of an every-other-day drinking paradigm but had no effect in mice with a history of chronic alcohol consumption (Erickson et al., 2021). The escalation in consumption during those first drinking sessions was reduced in mice expressing PMCA in mPFC astrocytes (Erickson et al., 2021). A recent study found that mice expressing PMCA in ventral striatal astrocytes had increased preference for low ethanol concentrations during an ethanol conditioned place preference task and showed increased ethanol consumption despite the presence of a bitter tastant (Peyton et al., 2025).

In the current study, there was a modest but significant increase in drinking in CIE exposed male LCK-GFP control mice while Air treated male mice of both viral groups showed a consistent reduction in drinking over the last four weeks of testing. The small but significant decrease in alcohol consumption in male Air treated mice was unexpected given that the groups were counterbalanced into Air or CIE groups based on baseline alcohol consumption levels. It is unlikely that these drinking effects were due to an environmental factor as female mice were also housed in the same room and there were no changes in female drinking levels over the course of the study. In addition, the drinking data was collected from 3 separate cohorts of mice using the same protocol over a span of a year. Nonetheless, CIE exposure increased alcohol consumption in male mice and this was blocked by PMCA expression in OFC astrocytes. CIE exposure did not increase drinking in female mice consistent with previous literature (Zamudio et al., 2021; Jury et al., 2017; Morales et al., 2015).

## 5. Conclusion

This study is the first to investigate how manipulating OFC astrocytes affects the ability of CIE exposure to alter OFC neuron excitability, astrocyte structure, and alcohol consumption in male and female mice. The findings of the study show that expressing the calcium exporter PMCA in OFC astrocytes produced sex-specific changes in neuronal physiology, astrocyte synaptic interaction, and drinking that varied depending on the prior alcohol history of the animal. These data provides additional evidence of the important role of astrocytes in mediating responses to ethanol.

## Figures and Tables

**Table 1:** Action potential kinetics and membrane properties of OFC neurons in LCK-GFP and PMCA male and female mice following air or CIE exposure.

MALES	LCK-GFP Air	LCK-GFP CIE	PMCA AIR	PMCA CIE
Amplitude (mV)	73.87 ± 3.40	72.61 ± 2.76	69.50 ± 2.17	65.06 ± 2.83
Threshold (mV)	−40.32 ± 1.62	−38.30 ± 1.13	−38.97 ± 1.27	−37.15 ± 1.54
Rise Time (ms)	0.68 ± 0.051	0.59 ± 0.034	0.69 ± 0.022	0.71 ± 0.045
Decay Time (ms)	1.94 ± 0.036	1.97 ± 0.029	1.93 ± 0.018	1.94 ± 0.034
Half-Width (ms)	2.16 ± 0.18	2.083 ± 0.11	2.12 ± 0.079	2.19 ± 0.11
mAHP (mV)	−11.57 ± 1.12	−12.17 ± 0.80	−11.60 ± 0.53	−10.95 ± 1.21
Rheobase (pA)	91.43 ± 8.25	82.50 ± 6.96	95.00 ± 7.32	97.50 ± 10.31
Input Resistance (MΩ)	82.54 ± 8.12	84.62 ± 6.72	90.40 ± 9.78	78.23 ± 8.10
Membrane Capacitance (C_m_)	14.02 ± 0.95	11.69 ± 0.87	10.63 ± 0.48^##^	11.62 ± 0.55
FEMALES	LCK-GFP Air	LCK-GFP CIE	PMCA AIR	PMCA CIE
Amplitude (mV)	71.39 ± 2.98	72.64 ± 2.31	69.30 ± 2.46	74.72 ± 3.38
Threshold (mV)	−42.47 ± 1.78	−40.57 ± 1.32	−41.37 ± 1.86	−41.23 ± 2.21
Rise Time (ms)	0.78 ± 0.048	0.72 ± 0.056	0.74 ± 0.035	0.76 ± 0.051
Decay Time (ms)	1.93 ± 0.044	1.96 ± 0.040	2.02 ± 0.020	1.99 ± 0.032
Half-Width (ms)	2.34 ± 0.160	2.14 ± 0.13	2.32 ± 0.10	2.41 ± 0.15
mAHP (mV)	−9.26 ± 0.997	−10.90 ± 1.04	−11.37 ± 0.88	−9.75 ± 1.17
Rheobase (pA)	100.00 ± 7.80	74.12 ± 6.36*	77.50 ± 8.55	111.54 ± 14.09*^#^
Input Resistance (MΩ)	85.66 ± 8.012	80.69 ± 6.71	103.35 ± 8.89	62.55 ± 7.21**
Membrane Capacitance (C_m_)	12.58 ± 1.37	12.51 ± 1.02	13.87 ± 0.99	13.99 ± 1.43

Action potential data (mean ± SEM) were analyzed using a two-way ANOVA with Holm-Sidák’s multiple comparisons post-hoc test. Symbols (*) and (**): p < 0.05 and p < 0.01 (Air vs. CIE); Symbols (^#^) and (^##^): p < 0.05 and p < 0.01, (LCK-GFP vs. PMCA).

## Data Availability

Data will be made available on request.
